# Protein Levels of 16 Cytochrome P450s and 2 Carboxyl Esterases Using Absolute Quantitative Proteomics: CYP2C9 and CYP3A4 Are the Most Abundant Isoforms in Human Liver and Intestine, Respectively

**DOI:** 10.3390/ph18121789

**Published:** 2025-11-25

**Authors:** Alexia Grangeon, Matthew L. Arwood, David Thacker, Fleur Gaudette, Jacques Turgeon, Veronique Michaud

**Affiliations:** 1 Centre de Recherche du Centre Hospitalier de l’Université de Montréal (CRCHUM), 900 St. Denis Street, Montréal, QC H2X 0A9, Canada; alexia.grangeon.chum@ssss.gouv.qc.ca (A.G.); fleur.gaudette.chum@ssss.gouv.qc.ca (F.G.); 2 GalenusRx Inc., P.O. Box 560025, Montverde, FL 34756, USA; mlarwood@galenusrx.com (M.L.A.); dthacker@galenusrx.com (D.T.); jturgeon@galenusrx.com (J.T.); 3 Faculty of Pharmacy, Université de Montréal, 2940 Chemin de la Polytechnique, Montréal, QC H3T 1J4, Canada

**Keywords:** CYP450, human hepatocytes, human liver microsomes, human intestinal mucosa, proteomics, cryopreserved human intestinal mucosa, pharmacokinetic-pharmacodynamic modeling

## Abstract

**Background/Objectives**: Metabolic enzymes are crucial for the detoxification of exogenously administered drugs, especially enzymes expressed in the intestine and the liver. Recent advancements in analytical methodologies enable sensitive and specific quantitative measurements of proteins, facilitating a more accurate evaluation of their expression and relative contribution to drug metabolism. **Methods**: The aim of the study was to characterize the protein expression levels of 16 Cytochrome P450s (CYP450s) and 2 carboxylesterases (CESs) in human liver and intestinal tissues using absolute quantification by HPLC-MS/MS. Human hepatocytes (HHEP) and human liver microsomes (HLM) were utilized, along with a novel intestinal preparation from cryopreserved human intestinal mucosa (CHIM), to perform proteomic analyses. **Results**: A comprehensive evaluation of 16 CYP450s and 2 CES enzyme expression in human liver and intestinal tissues is provided to reflect their relative abundance. Among the various in vitro systems evaluated, 14 of 16, 15/16, and 7/16 CYP450 of the isoforms analyzed were detected in HHEP, HLM, and CHIM, respectively. In hepatic systems, CYP2C9 exhibited the highest expression among CYP450 isoforms, a trend consistently observed in both HHEP and HLM. CYP3A4 was the most abundantly expressed isoform in CHIM preparations. Across all systems tested, CES1 and CES2 showed the highest overall protein expression levels, surpassing those of the CYP450s. **Conclusions**: Our findings demonstrate that the absolute quantification method employed is reliable, producing consistent results across two different in vitro hepatic systems (HHEP and HLM). This study supports the utility of absolute quantification approaches for accurately profiling drug-metabolizing enzymes and provides new, valuable insights to improve in vitro/in vivo extrapolation and more informed predictive pharmacokinetic modeling strategies.

## 1. Introduction

The intestine and the liver are largely involved in the metabolism of drugs [1,2]. Upon oral administration of a drug, the first metabolic enzymes that are encountered are localized in the intestine. Then, through the portal vein, drugs are presented to hepatocytes, where penetration in these cells is eased by the sinusoidal circulatory system. Many metabolizing enzymes, including the superfamily of cytochrome P450 (CYP450) and carboxylesterase (CES), are found in the intestine and in the liver [3,4]. Determination of their precise protein expression levels is mandatory while processing pharmacokinetic–pharmacodynamic models. In this study, we employed proteomic analyses to quantify drug metabolizing enzyme proteins expressed in the human liver and intestine. As protein abundance closely reflects cellular activity, such analyses offer a more direct measure of functional capacity than genomic or transcriptomic data alone.

Cryopreserved human intestinal mucosa (CHIM) is a novel system that allows for interrogation of metabolizing enzymes in different sections of the gut [5]. CHIM can be subdivided into the duodenum, proximal jejunum, medial jejunum, distal jejunum, and ileum. Studies have shown that CYP3A is the most active CYP450 in the duodenum [5,6]. However, there are only a few studies that interrogate the absolute amount versus the activity of metabolizing enzymes in the gut [7,8].

Human liver microsomes (HLMs) and human hepatocytes (HHEPs) are the main in vitro hepatic systems used in drug discovery to evaluate many pharmacokinetic parameters, such as partial metabolic clearances by specific enzymes [9,10,11,12]. However, these two systems are very different. HLMs are representative of the endoplasmic reticulum, obtained from homogenized liver, and are mainly used to evaluate drug metabolism by phase I enzymes [12,13]. HHEPs maintain the integrity of the cellular membrane barrier, are structurally rich in endoplasmic reticulum, are the major liver cell type, and account for approximately 80% of the total number of liver cells [14,15]. These cells allow the evaluation of the whole cellular metabolism, including phase I and phase II. In the literature, HLMs and HHEPs are often used and compared to the whole proteome or to study subcellular fractions. However, there is a lack of quantitative comparison of specific CYP450 and CES isoform expression in HLMs and HHEPs [16,17,18].

HHEPs and HLMs have been used to determine mRNA levels, protein expression, and activity for metabolic enzymes [19,20,21]. However, the scientific literature contains a lot of information where the whole proteome has been used with techniques that lack specificity, such as non-specific immunoassays and Western blotting. [14,16,18,19,20,21,22]. High-performance liquid chromatography/tandem mass spectrometry (HPLC-MS/MS) methods are becoming more popular in the proteomics field to quantify proteins because they have more precision and specificity than previously used methods. These methods are based on the quantification of proteotypic peptides following tryptic digestion, with the addition of stable isotope-labeled peptides as internal standards [23]. Many groups have developed absolute quantification methods to measure CYP450s in HLMs [4,19,20,24,25,26,27,28,29], but few have studied CYP450s in HHEPs [18,30]. Furthermore, there is a paucity of absolute quantification methods in both hepatic systems for CES [31].

Previously, we developed and validated an absolute quantification method for the analysis of 16 CYP450 isoforms by HPLC-MS/MS in several different human matrices, such as small intestine, heart, kidney, and liver [23,32]. The objectives of this study are to characterize the expression levels of 16 major CYP450s and 2 CESs in human liver and intestine (CHIM) using an absolute protein quantification by HPLC-MS/MS, and to compare expression trends across two different sources of hepatic in vitro systems (HHEPs and HLMs) commonly used in the context of drug metabolism studies. These findings aim to enhance our understanding of CYP450 isoform and CES protein distributions to improve in vitro/in vivo pharmacokinetic–pharmacodynamic modeling.

## 2. Results

### 2.1. Patient Demographics

Our study utilized many unique patient samples with a sizeable age range. The individual HHEPs (HHEP1 to 20) had a mean age of 47 (range 22–71); a total of 85% were Caucasian, and three individuals were Hispanic (*N* = 1), Asian (*N* = 1), or African American (*N* = 1), respectively (Table 1). For the pooled samples, the age range was 7–67 (HHEP21), 27–67 (HHEP22), and 16–69 (HHEP23). Caucasian was the main ethnicity among these pooled samples: 90% (HHEP21), 80% (HHEP22), and 85% (HHEP23) (Table 1). Genotyped HLMs (HLM1-HLM16) were represented by the following: a total of 41% were women, the mean age was 49 (range 17–83), and most were Caucasian (20) (Table 1). For the genotyped HLM samples, pharmacogenetics data was available for CYP2C9, CYP2C19, CYP2D6, and CYP3A5 (Supplementary Table S1).

For the CHIM samples, demographic information was available for three out of the four donors: of these, two were females, the age range was from 38 to 59, and the ethnicities included two Caucasian donors and one American Samoan (Table 1).

### 2.2. CYP2C9 Is the Most Expressed CYP450 in Two Different Hepatic Systems

Except for CYP1A1 and CYP1B1, all CYP450 proteins analyzed were detected and measured in some or all individual HHEPs. Of the 14 CYP450 isoforms found, all were detected in all individual HHEP donors, except for CYP2D6 and CYP3A7, which were present in 95% and 80% of donors, respectively (Table 2A).

**Figure 1:**
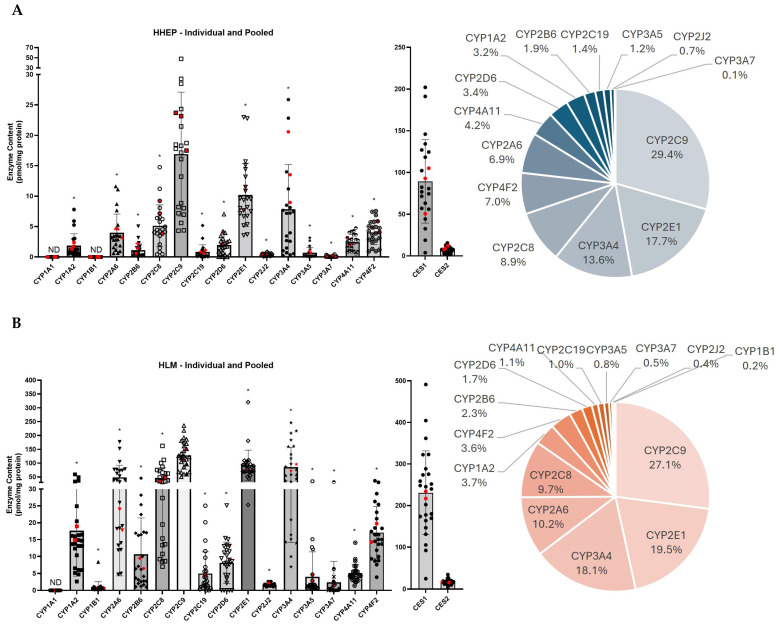
Quantitative analysis and comparative expression of CYP450 and CES protein levels in liver hepatocytes and liver microsomes. CYP2C9 is the most expressed CYP450 in human hepatocytes and liver microsomes: (**A**) Left—Human hepatocytes (HHEP) were processed and the metabolism enzyme content was analyzed. The enzyme content reported is calculated by using the absolute individual enzyme content (pmol) divided by total CYP450 or CES content (mg protein) for the corresponding sample. The black dots represent the individual HHEP samples, and the red dots represent the pooled HHEP samples. Right—The pie chart represents the percentage of CYP450 pmol/mg protein per total CYP450. (**B**) Left—Human liver microsomes (HLM) were processed in the same way as in A. The black dots represent the individual HLM samples, and the red dots represent the pooled HLM samples. Right—The pie chart represents the percentage of CYP450 pmol/mg protein per total CYP450. Data are displayed as mean ± standard deviation. ND = not detected. CYP protein levels were analyzed using CYP2C9 as the reference group: * indicates *p* < 0.0005. Also, see Table 2A,B.

The three pooled HHEP sample values are displayed in red in Figure 1A, and their values show a similar distribution compared to the individual sample values. In HHEP, CYP2C9 was the most abundant isoenzyme (29.4% of total CYP450 content) followed by CYP2E1 (17.7%) and CYP3A4 (13.6%) (Figure 1A). CYP2C8, CYP4F2, and CYP2A6 were moderately expressed in HHEP, each accounting for 6–9% of total expression, whereas CYP4A11, CYP2D6, CYP1A2, CYP2B6, CYP2C19, and CYP3A5 showed lower expression levels, ranging from 1 to 5%. CYP2J2 and CYP3A7 were the lowest expressed proteins (<1%). CYP1A1 and CYP1B1 were not detected in the HHEP samples, which is consistent with the results observed in iHHEP. CES1 and CES2 were expressed in all donors tested. CES1 (89.4 pmol/mg prot) was expressed much higher than CES2 (7.6 pmol/mg prot) (Figure 1A).

Quantitative proteomic analysis revealed no detectable CYP1A1 protein in HLMs, consistent with findings in HHEPs. In HLM samples, some CYP450 isoenzymes were not found in all donors. Specifically, CYP2D6 and CYP3A7 were found in 79% (19/24) and 75% (18/24) of HLM donors, respectively (Table 2B).

Three isoenzymes accounted for ~65% of total CYP450 content in HLMs: CYP2C9 (27.1%), CYP2E1 (19.5%), and CYP3A4 (18.1%) (Figure 1B). Two isoenzymes, CYP2A6 and CYP2C8, exhibited similar expression levels and together accounted for approximately 20% of the total CYP450 expression. CYP1A2, CYP4F2, CYP2B6, CYP2D6, CYP4A11, and CYP2C19 were moderately expressed, each accounting for 1–4% of the total CYP450 expression, whereas CYP3A5, CYP3A7, CYP2J2, CYP1B1, and CYP1A1 exhibited low expression levels (<1%) (Figure 1B). High interindividual variabilities were observed for CYP2C19, CYP2D6, and CYP3A7. The interindividual variability in CYP3A7 expression among HLM donors was primarily driven by a single donor, HLM011, who exhibited markedly elevated CYP3A7 levels (31.4 pmol/mg prot) compared to the average expression in the remaining HLM donors (1.2 pmol/mg prot) (Figure 1B and Table 2B). CES1 and CES2 were found in all 26 donors with a mean CES1 expression of 230.8 pmol/mg protein and a mean CES2 expression of 16.6 pmol/mg protein (Figure 1B).

**Figure 2:**
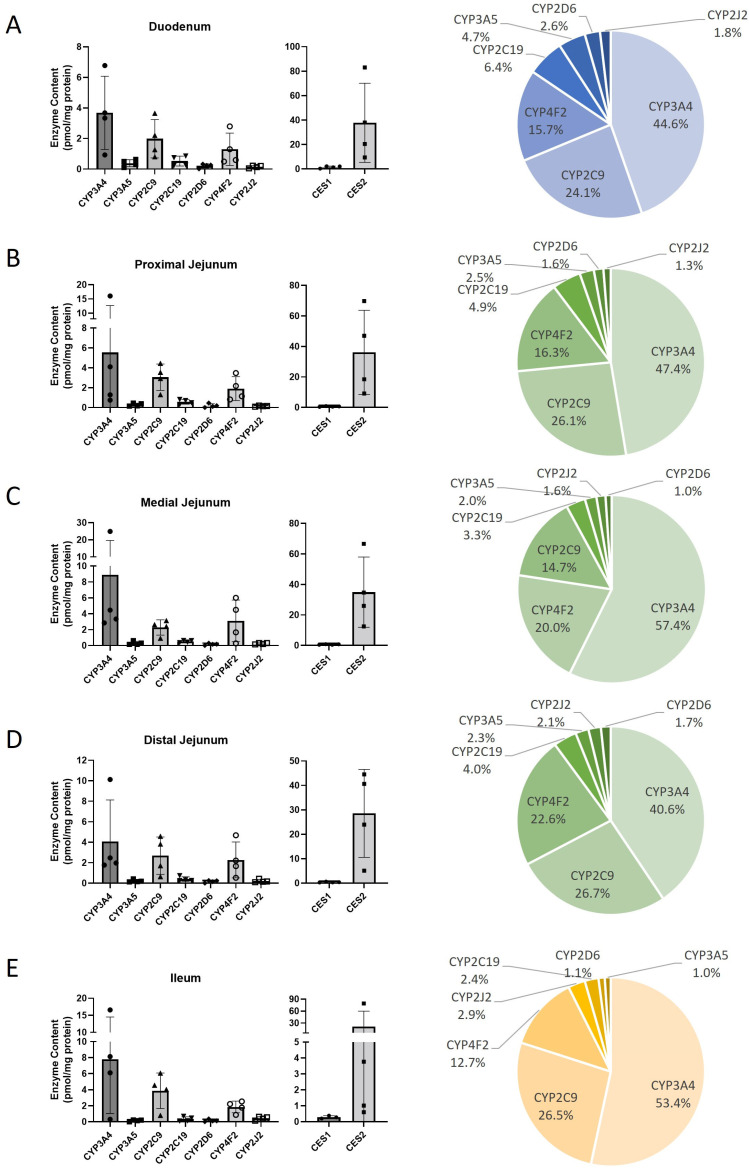
Quantitative analysis of CYP450 and CES protein levels in cryopreserved human intestinal mucosa (CHIM). CYP3A4 is the most expressed CYP450 isoform in the intestine: Left—Cryopreserved human intestinal mucosa (CHIM) sections were processed and the enzyme content was analyzed. (**A**) Duodenum, (**B**) proximal jejunum, (**C**) medial jejunum, (**D**) distal jejunum, and (**E**) ileum enzyme content was calculated using the absolute individual enzyme content (pmol) divided by total CYP450 or CES content (mg protein) for the corresponding sample. Right—The pie chart represents the percentage of CYP450 pmol/mg protein per total CYP450. Data are displayed as mean ± standard deviation. Also, see Table 2C and Supplementary Figure S1.

CHIM samples were divided into five distinct intestinal sections: duodenum, proximal jejunum, medial jejunum, distal jejunum, and ileum. Across all five sections, only 7 out of the 16 analyzed CYP450 isoforms were expressed—namely, CYP2C19, CYP2C9, CYP2D6, CYP2J2, CYP3A4, CYP3A5, and CYP4F2. Hence, CYP1A1, CYP1A2, CYP1B1, CYP2A6, CYP2B6, CYP2C8, CYP2E1, CYP3A7, and CYP4A11 were not detected in any of the sections (Table 2C). There were no major differences across the five tissue sections in their relative expression patterns (Figure 2). Notably, three isoforms—CYP3A4, CYP2C9, and CYP4F2—accounted for the majority of total CYP450 expression, contributing approximately ~80–90%. When the CYP450s are compared across each of the five sections there are no significant differences in enzyme protein levels (Supplementary Figure S1). In contrast to HHEPs and HLMs, where CES1 is the dominant CES expressed (Table 2A,B), CES2 was expressed at much higher levels than CES1 in CHIM, 21.1–37.7 pmol/mg protein and 0.29–1.51 pmol/mg protein, respectively (Table 2C).

### 2.3. Protein Expression and Enzyme Activity Are Correlated in Most Liver Samples

Enzyme activities for HHEPs and HLM01 to HLM16 were determined by Thermo Fischer Scientific and Xenotech for each sample used in our analyses. In both pooled and individual HHEP samples, enzymatic activity was measured and provided for nine CYP450 isoforms: CYP1A2, CYP2A6, CYP2B6, CYP2C8, CYP2C9, CYP2C19, CYP2D6, CYP2E1, and CYP3A4/5. Enzymes activities ranged as follows: CYP3A4/5 > CYP2C9 > CYP2C8 > CYP2E1 > CYP1A2 > CYP2B6 > CYP2D6 > CYP2A6 > CYP2C19 (Supplementary Figure S2A). All CYP450 activities were obtained in all donors. High correlations between CYP450 content and activities were observed for the CYP1A2, CYP2A6, CYP2B6, CYP2C8, CYP2C19, and CYP2D6 (r = 0.70–0.80), whereas CYP3A4 showed moderate correlation (r = 0.53) and CYP2C9 and CYP2E1 were not correlated with enzymes activities (r = 0.29–0.38). Correlation analyses obtained with Spearman’s correlation analyses are reported in Table 3A and Supplementary Figure S2B.

In HLMs, the CYP450 pattern of activity was similar to the one measured in HHEPs (CYP4A11 was assessed exclusively in HLM samples). Enzymes activity levels followed the following order: CYP2E1 > CYP3A4/5 > CYP2C9 > CYP2C8 > CYP4A11 > CYP2A6 > CYP2B6 > CYP1A2 > CYP2D6 > CYP2C19 (Supplementary Figure S3A). In contrast to HHEPs, all CYP450 enzyme contents in HLMs showed significant correlations with their respective enzymatic CYP450 activities (r = 0.65–0.98) (Table 3B and Supplementary Figure S3B).

In CHIM, a significant correlation between protein levels and enzyme activity was observed only for CYP2C19 (r = 0.70) and CYP2D6 (r = 0.58) (Table 3C, Supplementary Figure S4). CYP2C9, CYP3A4, CYP2J2, and CES2 showed weak correlations between protein levels and corresponding enzyme activity.

### 2.4. The CYP450 Profiles in Two Different Hepatic Systems Demonstrate High Correlation

**Figure 3:**
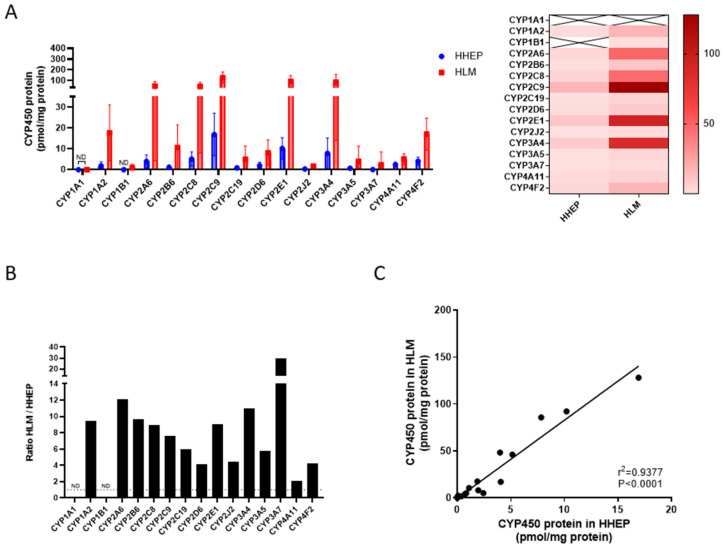
Comparative correlation of CYP450 isoform profiles in hepatocytes and microsomal preparations. The CYP450 profiles in HHEPs and HLMs correlate strongly: (**A**) Left—The CYP450 mean protein contents from HHEPs and HLMs are re-graphed together (same data as Figure 1A,B left) for comparison. Right—The CYP450 mean protein content data (from Figure 1A,B) are represented in a heat map chart. (**B**) The ratio of CYP450 protein content of HLM/HHEP was calculated. (**C**) The average CYP450 protein contents from HLMs and HHEPs were plotted in a scatter plot, and correlation analysis was performed. In Figure 2A-left, data are displayed as mean ± standard deviation. ND = not detected. A simple linear regression (**C**) was performed for statistical analysis. Also, see Table 2 and Table 3.

In total, the measurement of 16 CYP450 isoforms was attempted in both hepatic cells and liver microsomes (pooled + individual). All were detected except for CYP1A1, which was not detected in either of the tested hepatic systems, and for CYP1B1, which was observed only in HLMs (Figure 3A). The concentration levels of all CYP450 isoforms were higher in HLMs compared to HHEPs, which was expected due to the enriched nature of HLM preparations (Figure 3A and Table 2A,B). The differences in CYP450 levels between hepatic systems were protein dependent, as the HLM-to-HHEP ratio is illustrated (Figure 3B). The HLM-to-HHEP ratio was low (≤3) for CYP1B1 and CYP4A11, moderate (4–6) for CYP4F2, CYP2J2, CYP2D6, CYP3A5, and CYP2C19, moderate-high (7–10) for CYP2C9, CYP2E1, CYP1A2, CYP2C8, and CYP2B6, and high (≥10) for CYP3A4, CYP2A6, and CYP3A7. The mean protein levels of the CYP450s were highly correlated between HLMs and HHEPs (Figure 3C, r^2^ = 0.938, *p* < 0.0001).

## 3. Discussion

Human liver microsomes are widely used in enzyme kinetics and drug metabolism studies due to their high and consistent CYP450 content. Compared to hepatocytes in suspension, HLMs are more readily available, easier to handle in practice, and offer greater experimental reproducibility [12]. Hepatocyte systems are preferred for more comprehensive metabolism studies, including phase I and II metabolism, and induction studies. Nevertheless, both hepatocytes and microsome systems are used to measure drug metabolism in the liver and for in vitro studies. Multiple groups have compared these systems for a larger group of proteins, but there is a lack of CYP450 absolute amount comparison for these systems in the literature [16,17,18]. Additionally, the CHIM system is novel, and there is not much literature published exploring this system.

Our patient sample was predominantly composed of Caucasian donors. This demographic distribution may introduce certain limitations and potential biases, affecting the generalizability of our findings to more diverse global populations (Table 1). However, our study included a substantial number of HHEP donors (n = 60), spanning a wide age range (7–71 years), and an ideal male-to-female ratio (HHEP 50%: 50%). These statistics represent the robustness of our data and demonstrate that our data can be used as a reference for further studies.

The distribution pattern of CYP450 protein isoforms in hepatocytes and liver microsomes was highly similar (Figure 1). In both systems, CYP2C9 was the most abundantly expressed isoform, accounting for 27–29% of total CYP450 content, followed by CYP2E1 (18–19%) and CYP3A4 (13–18%) (Figure 1). These findings differ from the revised human liver CYP450 profile published by Michaels et al., which, based on liver microsomes data, indicated that the CYP3A family was the most highly expressed, followed by the CYP2C and CYP4F families [20]. However, their analysis did not employ absolute quantification for all CYP450 isoforms: only the CYP4F family was quantified directly, while the remaining enzymes were measured using immuno-quantification methods. In our analysis, CYP4F2 was highly expressed (4–7% of the total pie chart) and was in the top six most expressed CYP450s for all hepatic systems [20,21]. Drodzik et al. obtained a distribution similar to ours in human liver microsomes, except for CYP2E1, which was more expressed (22–37%) [24,25]. In their analyses, Drozdzik et al. quantified only nine CYP450 isoforms using a single proteotypic peptide per enzyme, whereas our study utilized two peptides per enzyme, enhancing the accuracy and reliability of quantification [24]. Couto et al. observed similar CYP2E1 expression in liver microsomes (21%) and other CYP450 expression but a lower CYP2C9 level (15%) compared to our results (Figure 1) [26]. The other CYP450s displayed the same order of expression between hepatocytes and liver microsomes as our study: CYP2C8 ~ CYP2A6 > CYP4F2 > CYP1A2 > CYP4A11 ~ CYP2D6 ~ CYP2B6 > CYP2C19 ~ CYP3A5 > CYP2J2 ~ CYP3A7 [25,26]. CYP1B1 was not expressed (HHEPs) or had very low expression (HLMs), and CYP1A1 was not expressed in either hepatic system (Table 2). For the CYP450s measured by Drozdzik et al. and Couto et al., a similar expression pattern was observed in comparison to our findings [24,26].

A meta-analysis by Achour et al. reported CYP450 quantification values from HLM samples, independent of the analytical methods used [33]. Of the 50 studies included for comparison, only 5 employed absolute quantification techniques, and many focused on quantifying only one or two CYP450 isoforms [4,19,27,28,29]. Consistent with our findings, the reported CYP450 abundances were generally within a similar range, with the exception of CYP3A4, which was identified as the most abundantly expressed isoform in their meta-analysis [33]. As reported by Drozdzik et al., Couto et al., and corroborated by our findings, CYP3A4 does not appear to be the most abundantly expressed CYP450 isoform in HLM but rather ranks as the third most expressed [25,26].

A paucity of data for a few CYP450s in HHEPs is published as it pertains to their relative expression [18,30]. In a study similar to ours, Vildhede et al. compared isolated hepatocytes and liver tissue. These authors reported a high correlation and comparable CYP450 expression levels between the two systems (Figure 3) [30]. However, their study did not provide absolute quantification data. At a protein level, CYP2C9 seemed to be the most abundantly expressed isoform, followed by CYP3A4, CYP2A6, CYP2E1, and CYP2C8. Other CYP450s were also in a similar order of expression. Comparison of our results with the existing literature is somewhat challenging, as most data reported from liver microsomes and human hepatocytes have been generated using less specific methodologies, such as immuno-quantification, mRNA expression analysis, or enzyme activity assays. Therefore, our findings provide valuable and more precise insights that can contribute meaningfully to the current knowledge [19,20,21].

CYP450 protein abundance was very well correlated between HLMs and HHEPs in our study (r^2^ = 0.938), which is in line with our observation of similar enzyme distribution for both hepatic systems (Figure 3C). Although CYP450 protein expression between the two systems was highly correlated, their functional expression (enzymatic activity) showed weaker correlation. This discrepancy was particularly evident for CYP2C9 and CYP2E1 in HHEPs. The reduced correlation can be attributed to factors such as ER enrichment, differences in the incubation protocols, lower turnover of CYP2C9 and CYP2E1 in HHEP than HLM due to cell integrity and/or drug uptake (passive diffusion or transporters). Our data also showed a higher level of expression for all CYP450 in HLMs compared to human hepatocytes, which is consistent with data from Wegler et al., who also showed increased protein in HLMs versus hepatocytes [18] (Figure 3A). The HLM-to-HHEP ratio ranged from 2.1 (CYP4A11) to 29.7 (CYP3A7) in our data, whereas Wegler et al. found a ratio ranging from 1.2 (CYP2C9) to 46 (CYP2C19) [18] (Figure 3B). These authors only measured 11 CYP450s, so the comparison could not be made for all CYP450s. Furthermore, their average enrichment ratio between HLMs and HHEPs was slightly lower than what we observed, though still comparable. In contrast to our findings for CYP2C19, which showed a ratio of 6 (Figure 3B), they reported a substantially higher concentration of CYP2C19 in HLMs, which explained the high enrichment ratio they observed (Wegler et al., ratio of 46). However, the metabolic activity and protein amount did not correlate well with CYP2C19 in HLMs (r = 0.32) compared to HHEPs (r = 0.88), suggesting that the high concentration observed in HLMs might not be specific to CYP2C19. In our study, the correlation between metabolic activity and CYP450 absolute quantification in HHEPs was variable within the CYP450s analyzed; no correlation was observed for CYP2C9 and CYP2E1, but a high correlation was observed for CYP2B6 and CYP2D6 protein (Table 3A). For HLMs, we observed a high correlation between metabolic activity and absolute quantification for all CYP450s, whereas Wegler et al. observed a low correlation for CYP2C19, CYP2C9, CYP2B6, and CYP2D6 [18] (Table 3B). However, they did not use absolute quantification methods and isotope-labeled peptides as internal standards as in our study; their less specific methods could explain the differences observed. Altogether, metabolic activities and absolute quantifications were well correlated for the two hepatic systems in our study (Table 3A,B).

The good correlation observed between protein abundance and enzyme activity in HHEPs and HLMs, presented in Table 3A and Table 3B, did not extend to CHIM samples (Table 3A, Supplementary Figures S2–S4). Among the seven enzymes analyzed, only two—CYP2C19 (r = 0.70) and CYP2D6 (r = 0.58)—demonstrated statistically significant correlations (Table 3C). Notably, the three most highly expressed enzymes across all intestinal sections—CES2, CYP3A4, and CYP2C9—showed no correlation between protein expression and enzymatic activity (Table 3C, Figure 2). This stands in stark contrast to hepatic systems, where seven out of nine enzymes in HHEPs and all ten enzymes in HLMs exhibited significant correlations with enzyme activity (Table 3A,B).

The weak correlation between CYP2C9 and CYP2E1 protein abundance and activity in HHEP, compared to strong correlation observed in HLM, likely reflects differences between intact hepatocytes and microsomal preparations which appear substrates and/or enzyme dependent. Plausible explanations may include the following: in HHEP cryopreservation and thawing may subtly affect enzyme functionality without altering protein levels. Intact cells maintain complex intracellular compartments, where CYP proteins may be mislocalized or lack essential cofactors, reducing catalytic efficiency despite measurable protein levels. Additional mechanisms include dynamic enzyme conformational states, non-steady-state metabolism, and competing metabolic pathways such as conjugation reactions of 4-OH-diclofenac (possible in HHEP but absent in microsomal systems due to incubation conditions). Finally, drug access to CYP450 enzymes is restricted by cellular membranes and efflux/influx transporters, particularly for hydrophilic and acidic substrates like CYP2E1 and CYP2C9 substrate probes, used in these assays. In contrast HLM provide direct substrate access. Collectively, these factors may contribute to the observed discrepancy between protein and activity in HHEP. 

Similar factors may explain weak correlations for highly expressed isoforms in the intestine (CHIM) where active drug transport is substrate-selective and can markedly reduce intracellular concentrations of probe substrates, especially for CYP3A4, leading to poorer alignment between activity and expression. The physicochemical properties of CYP2C9 substrates may limit their transmembrane distribution at physiological pH, then decrease their intracellular concentrations and, indirectly, their metabolism (perceived enzymatic activity). Highly expressed enzymes are often regulated by multiple pathways; this complexity increases susceptibility to modulation such as post-translational modifications, mislocalization, proper folding, and consequently could contribute to discrepancies between protein levels and activity.

The reduced number of detectable metabolic enzymes in CHIM observed in our study is consistent with findings by Ahire et al., who also reported fewer proteins in CHIM compared to HLMs (Table 2C) [8]. Similarly, Krogstad et al. found a greater number of active CYP450 enzymes in the liver (7/7) compared to the intestine (4/7) [34]. To our knowledge, there is a lack—or at most, very limited—in the published literature correlating enzyme levels to activity in CHIM outside of our report in Table 3C.

In two independent studies, Li et al. ranked CYP450 enzyme activity in CHIM models [5,6]. In the smaller study (CHIM, n = 4), CYP3A4 activity was predominant, accounting for over > 75% of the normalized total activity, while CYP2C9 and CYP2J2 contributed less than 15% of the normalized activity [5]. In our study, the three most expressed CYP450 enzymes in CHIM were CYP3A4, CYP2C9, and CYP4F2 (Table 2C). Li et al. did not assess CYP4F2 levels in their CHIM model. In their larger study (CHIM, n =10), they reported similar findings to their earlier work, with CYP3A activity dominating, and CYP2J2 and CYP2C9 ranking as distant second and third contributors to total CYP450 activity [6]. These findings underscore the significant role of CYP3A4 in intestinal CYP450-mediated metabolism. Our data, based on total protein quantification, demonstrate that CYP3A4, CYP2C9, and CYP4F2 are the most abundantly expressed in the CHIM medium; CYP2J2 is expressed at a very low level. This aligns with the findings of Li et al., who reported a predominant role for CYP3A4 activity (Figure 2).

CES1 and CES2 were both detected in HHEPs and HLMs, exhibiting similar distribution patterns (Figure 1). CES1 was more highly expressed than CES2 in both hepatic systems, which is consistent with previous reports in the literature (Figure 1 and Figure 2) [3,22,31]. Quantitative studies on CES expression in HLMs or hepatocytes are limited, and to our knowledge, Sato et al. is the only group that has quantified CES levels in HLMs using HPLC-MS/MS [31]. CES1 expression obtained by Sato et al. in HLMs ranged from 171 to 801 pmol/mg in 16 individual donors and from 16 to 57 pmol/mg for CES2 [31]. Our results showed slightly lower expression levels of CES1, ranging from 25 to 491 pmol/mg protein (Figure 1B). CES2 expression fell within a similar range, from 5 to 35 pmol/mg protein (Figure 1B). Notably, Sato et al. quantified CES using only a single peptide, whereas our analysis employed two peptides, enhancing both the accuracy and specificity. They observed low expression levels in human liver cytosol, as we observed low expression in human hepatocytes [31]. CES1 and CES2 expression levels in hepatocytes were approximately 2.5-fold lower than those observed in HLMs (Figure 1A and Figure 2).

Interestingly, CES2 was the predominant isoform in the intestinal system, whereas CES1 was dominant in HHEPs and HLMs (Figure 1 and Figure 2). This observation aligns with findings from Ahire et al. [8] and Basit et al. [7], who also reported CES2 as the major isoform in the intestine and CES1 as predominant in the liver.

## 4. Materials and Methods

### 4.1. Chemicals and Reagents

HPLC-MS-grade water (H_2_O) was purchased from EMD Millipore (Billerica, MA, USA). HPLC-MS-grade acetonitrile (ACN), MS-grade trypsin protease, dithiothreitol (DTT), ammonium bicarbonate (NH_4_HCO_3_), Mammalian Protein Extraction Reagent (MPER), Tissue Protein Extraction Reagent (TPER), 10× Phosphate-Buffered Saline (PBS), and the Pierce^®^ BCA Protein Assay Kit were obtained from Thermo Fischer Scientific (Waltham, MA, USA). Formic acid (FA), iodoacetamide (IAA), trifluoroacetic acid (TFA), and bovine serum albumin (BSA) were purchased from Sigma Aldrich (St. Louis, MO, USA). Human hepatocytes (HHEP; 20 individuals and 3 pools), genotyped human liver microsomes (HLM1–16; 16 individuals), and optithaw hepatocyte media were purchased from Xenotech (Kansas City, KS, USA). Non-genotyped human liver microsomes (HLMs; 8 individuals and 2 ultrapools) were obtained from Thermo Fischer Scientific (Waltham, MA, USA) and Xenotech (Kansas City, KS, USA). Cryopreserved Human Intestinal Mucosa (CHIM; 4 individuals) was purchased from In Vitro ADMET Laboratories LLC (IVAL, Columbia, MD, USA). Proteotypic peptides and their stable isotope-labeled internal standards were synthesized by New England Peptides (Boston, MA, USA) [19]. All peptide purities were superior to 95%, and the concentration/net peptide content was determined by amino acid analysis.

### 4.2. Preparation of Human Hepatocyte and Intestinal Mucosa Lysate

HHEPs and CHIM were stored in liquid nitrogen until use. For experimental use, cryopreserved HHEPs and CHIM were removed from storage and immediately submerged in a warm water bath for approximately 80 s. Cell pellets were then transferred into the OptiThaw media tube, which was previously warmed in a water bath at 37 °C for 30 min. Samples were gently inverted and centrifuged at 100× *g* for 10 min at room temperature. Cell pellets were then resuspended with 1X PBS buffer and transferred to an Eppendorf tube for centrifugation at 500× *g* for 10 min at room temperature. Supernatants were discarded, and the PBS wash was repeated. Cell pellets from HHEPs and CHIM were resuspended with 300 µL of MPER buffer and 150 µL of TPER buffer, respectively, and vortexed gently for approximatively 5 s. The samples were sonicated for 5 min at 4 °C and then put at −80 °C for at least 1 h. Samples were then unfrozen at room temperature and centrifuged at 500× *g* for 10 min at 4 °C. The protein concentration of the samples was determined using a BCA Protein Assay Kit (Thermo Fischer Scientific; Waltham, MA, USA). Samples were kept at −80 °C until further analysis.

### 4.3. Subjects

Human hepatocytes (designated HHEP01 to HHEP20) were obtained from 20 individual donors (10 women and 10 men). HHEP samples were also obtained from 3 separate donor pools; these pooled donors were distinct from the individual HHEP donors used in the study. Two pooled hepatocyte preparations (designated HHEP21 and HHEP22) were generated from 10 donors each, with equal distribution of 5 females and 5 males per pool. A third pool (HHEP23) was prepared from 20 donors comprising 10 females and 10 males.

Genotyped human liver microsomes (designated HLM01 to HLM16) were obtained from 16 donors. Non-genotyped HLM samples (designated HLM17 to HLM26) were sourced from 8 individual donors and 2 UltraPool HLM preparations (150 donors per preparation).

CHIM samples were obtained from 4 individual donors. For each CHIM donor, 5 distinct sections of the small intestine were obtained: duodenum, proximal jejunum, medial jejunum, distal jejunum, and ileum.

CYP activities in HHEP and HLM were measured using validated probe substrates and protocols, and the results were provided by the supplier: the CYP450 enzyme marker substrate activity in pmol/mg protein/min values was obtained from the XenoTech and In Vitro ADMET Laboratories product information sheets.

### 4.4. LC-MS/MS-Based Protein Quantification

Our absolute quantification method was used to quantify 16 CYP450 (CYP1A1, CYP1A2, CYP2A6, CYP1B1, CYP2B6, CYP2C8, CYP2C9, CYP2C19, CYP2D6, CYP2E1, CYP2J2, CYP3A4, CYP3A5, CYP3A7, CYP4A11, and CYP4F2) and 2 CES (CES1 and CES2) proteins in human hepatocytes and liver microsomes. This proteomic approach is based on the quantification of two proteotypic peptides specific to each CYP450 [23]. Because endogenous peptides were present in all matrices, calibration standards were prepared by fortifying NH_4_HCO_3_ with the working solutions, yielding calibration curve ranges of 0.1 to 15 nM for CYP1A1, 1A2, 1B1, 2B6, 2C19, 2J2, 3A5, 3A7, 4F2, CES1 and CES2 and 0.1–50 nM for CYP2C8, 2C9, 2D6, 2E1 and 3A4. Quantification of CES1 and CES2 was incorporated as an extension of our previously published validated protein assay [23]. Qualifications of CES1 and CES2 were performed as follows. We used two proteotypic peptides for each CES, i.e., ELIPEATEK and YLGGTDDTVK for CES1 and ADHGDELPFVFR and IQELEEPEER for CES2. The assay was linear from 0.1 to 15 nM, and the LLOQ and intra/inter-day precisions were better than 10.0% and 14.9%, respectively; the LLOQ and intra/inter-day accuracies were 100.8–115.7% and 86.9–114.4%. According to current U.S. FDA guidelines for bioanalytical methods, our assay was also validated for selectivity, stability, and the matrix effect (Tables S2–S5). Detailed results and validation of the CES1 and CES2 isoform methods are described in Appendix A.

Peptides were obtained by tryptic digestion of proteins, with isotope-labeled peptides used as internal standards. As previously described, HHEPs, HLMs, and CHIM were prepared for proteomics analysis as follows: (1) dilution of proteins in NH_4_HCO_3_ (50mM, pH 7.8) to a concentration of 1 mg/mL (HLMs) or 3 mg/mL (HHEPs, CHIM); (2) reduction with dithiothreitol for 20 min at 60 °C; alkylation with iodoacetamide for 15 min at 37 °C. Samples were then incubated for 16 h at 37 °C, and digestion ended by acidifying samples with 20 µL of ACN:H_2_O:TFA (40:60:1, *v*/*v*) that included internal standards. The samples were centrifuged at 16,000× *g* for 10 min. Clear supernatant was evaporated to dryness, and the dried extract was resuspended with ACN:H_2_O:TFA (10:90:0.1, *v*/*v*).

The peptides obtained were separated by a Biobasic-8 100 mm × 1 mm, 5 µM analytical column (Thermo Scientific, Waltham, MA, USA) using a gradient mobile phase at a flow rate of 75 µL/min. The mobile phase consisted of A (0.1% FA in ACN) and B (0.1% FA in H_2_O). The gradient was as follows: 0 min (5:95, A:B, *v*/*v*); 1–61 min (linear gradient up to 32:68, A:B, *v*/*v*); 61 min (initial conditions); and 61–75 min (re-equilibration). The column temperature was set at 40 °C, and the injection volume was 2 µL. For each peptide, two or three Selected Reaction Monitoring (SRM) transitions were selected, and the final protein amount was calculated as the average of the two proteotypic peptide results for each CYP450 and CES protein.

The mass spectrometry proteomics data is available in the public repository PeptideAtlas SRM Experiment Library (PAASEL) ProteomeXchange with the data set identifier PASS05929.

### 4.5. Data and Statistical Analysis

Each CYP450 absolute protein amount was measured in duplicate and in 3 independent experiments. The final absolute quantification level was measured with two proteotypic peptides by calculating the mean of the CYP450 amount obtained with two peptides. GraphPad Prism Version 10.4 (GraphPad Software, La Jolla, CA, USA) was used to generate the Figures and perform statistical analyses. A one-way ANOVA analysis followed by Dunnett’s post-hoc test to compare each detectable CYP enzyme’s expression relative to CYP2C9 (reference group) was performed. Microsoft Excel was used to generate pie charts (Microsoft 365 Apps for business, Version 2401, Redmond, WA, USA). Correlation analysis between protein amount and activity was evaluated using Spearman’s correlation analysis, and a *p* value < 0.05 was considered statistically significant. Additionally, a simple linear regression analysis was performed to determine the r^2^ value for the displayed data.

## 5. Conclusions

Our method enabled the quantification of 15 out of 16 CYP450 isoforms and two CES isoforms across two key hepatic systems: human hepatocytes (HHEPs) and human liver microsomes (HLMs), with CYP2C9 being the major CYP450 isoform expressed in these systems. Additionally, our method successfully quantified nine metabolic enzymes in the CHIM system. Overall, the distribution patterns of CYP450 and CES enzymes were largely consistent between HHEPs and HLMs, although expression levels were generally lower in hepatocytes (as expected). This study demonstrates the utility of our absolute quantification approach for accurately profiling drug-metabolizing enzymes. The insights gained not only enhance our understanding of CYP450 enzyme distribution across hepatic systems but also contribute to improving in vitro/in vivo extrapolation and refining predictive pharmacokinetic–pharmacodynamic modeling strategies.

## Figures and Tables

**Table 1:** Demographic characteristics for the following: Human hepatocytes (HHEPs), human liver microsomes (HLMs), and cryopreserved human intestinal mucosa (CHIM). Abbreviations: M, Male; F, Female; C, Caucasian; AA, African American; H, Hispanic; A, Asian; AS, American Samoan; AN, Anoxia; HT, Head trauma; CVA, Cerebrovascular Accident; GSW, Gunshot wound.

Subject	Type	Sex	Age (Years)	Ethnicity	Cause of Death
HHEP01	Individual	M	52	C	AN
HHEP02	Individual	M	56	C	HT
HHEP03	Individual	F	59	C	CVA
HHEP04	Individual	F	48	C	CVA
HHEP05	Individual	F	28	AA	HT
HHEP06	Individual	F	71	C	CVA
HHEP07	Individual	F	22	C	CVA
HHEP08	Individual	F	70	C	CVA
HHEP09	Individual	F	56	C	AN
HHEP10	Individual	M	30	C	CVA
HHEP11	Individual	M	55	C	AN
HHEP12	Individual	M	45	C	AN
HHEP13	Individual	M	61	C	HT
HHEP14	Individual	M	31	H	AN
HHEP15	Individual	F	56	C	CVA
HHEP16	Individual	F	24	C	HT
HHEP17	Individual	F	56	C	CVA
HHEP18	Individual	M	43	A	AN
HHEP19	Individual	M	44	C	AN
HHEP20	Individual	M	41	C	HT
HHEP21	Pool	M (5), F (5)	7–67	AA (1), C (9)	AN (3), CVA (6), HT (1)
HHEP22	Pool	M (5), F (5)	27–67	AA (2), C (8)	AN (2), CVA (5), HT (3)
HHEP23	Pool	M (10), F (10)	16–69	AA (1), C (17), A (2)	AN (10), CVA (3), HT (7)
HLM01	Individual	M	44	H	AN
HLM02	Individual	F	78	C	CVA
HLM03	Individual	F	57	C	HT
HLM04	Individual	M	71	C	CVA
HLM05	Individual	M	21	C	HT
HLM06	Individual	F	62	C	AN
HLM07	Individual	M	49	C	HT
HLM08	Individual	M	45	C	CVA
HLM09	Individual	F	55	C	CVA
HLM10	Individual	F	49	C	AN
HLM11	Individual	M	41	C	HT
HLM12	Individual	F	48	C	CVA
HLM13	Individual	M	39	C	HT
HLM14	Individual	M	39	C	AN
HLM15	Individual	M	56	AA	CVA
HLM16	Individual	M	35	AA	CVA
HLM17	Individual	M	34	C	GSW
HLM18	Individual	F	44	C	CVA
HLM19	Individual	F	79	C	CVA
HLM20	Individual	F	17	C	AN
HLM21	Individual	M	48	C	CVA
HLM22	Individual	F	34	H	AN
HLM23	Individual	M	83	C	CVA
HLM24	Individual	M	51	C	HT
HLM25	Pool	UltraPool HLM 150			
HLM26	Pool	UltraPool HLM 150			
CHIM01	Individual	F	49	C	CVA
CHIM02	Individual	F	59	AS	CVA
CHIM03	Individual	M	38	C	HT
CHIM04	Individual	Unknown	Unknown	Unknown	Unknown

**Table 2:** Protein expression levels of 16 CYP450 and 2 CES in the following: (**A**) Individual and pooled human hepatocytes; (**B**) individual and pooled human liver microsomes; and (**C**) cryopreserved human intestinal mucosa. Protein amounts are expressed in pmol/mg protein. ND—not detected.

(A)																				
Individual HHEP										Pooled HHEP										
Protein		Mean ± SD			Min–Max		*N* (20)			Protein				Mean ± SD		Min–Max			*N* (3)	
CYP1A1		ND					20			CYP1A1				ND					3	
CYP1A2		1.88 ± 2.10			0.30–7.81		20			CYP1A2				1.79 ± 0.56		1.28–2.39			3	
CYP1B1		ND					20			CYP1B1				ND					3	
CYP2A6		3.95 ± 3.29			0.61–11.6		20			CYP2A6				4.23 ± 0.84		3.26–4.74			3	
CYP2B6		1.07 ± 1.24			0.08–5.05		20			CYP2B6				1.28 ± 0.77		0.51–2.06			3	
CYP2C19		0.82 ± 1.26			0.02–51.8		20			CYP2C19				0.85 ± 0.27		0.60–1.13			3	
CYP2C8		4.89 ± 3.53			0.42–14.8		20			CYP2C8				6.75 ± 2.71		3.84–9.22			3	
CYP2C9		16.2 ± 10.7			4.32–47.8		20			CYP2C9				21.5 ± 3.44		17.5–23.7			3	
CYP2D6		1.80 ± 1.71			0.00–7.03		20			CYP2D6				2.98 ± 1.07		2.24–4.21			3	
CYP2E1		10.2 ± 5.57			3.56–23.0		20			CYP2E1				10.0 ± 2.05		7.67–11.5			3	
CYP2J2		0.39 ± 0.22			0.067–0.85		20			CYP2J2				0.38 ± 0.11		0.25–0,46			3	
CYP3A4		6.85 ± 7.16			0.23–25.9		20			CYP3A4				14.4 ± 5.85		8.97–20.6			3	
CYP3A5		0.67 ± 0.95			0.034–3.16		20			CYP3A5				0.81 ± 0.25		0.64–1,10			3	
CYP3A7		0.07 ± 0.12			0.00–0.44		20			CYP3A7				0.13 ± 0.08		0.065–0,23			3	
CYP4A11		2.43 ± 1.36			0.52–4.74		20			CYP4A11				2.46 ± 0.62		2.0–3,16			3	
CYP4F2		3.91 ± 2.09			0.90–7.55		20			CYP4F2				4.93 ± 0.90		4.08–5.87			3	
Total CYP450		55.8 ± 4.40								Total CYP450				72.4 ± 6.04						
CES1		90.4 ± 52.9			3.84–202		20			CES1				82.8 ± 28.7		50.5–105.2			3	
CES2		7.27 ± 3.70			1.84–15.6		20			CES2				9.67 ± 2.45		7.78–12.4			3	
Total CES		97.7 ± 58.8								Total CES				92.5 ± 51.7						
(**B**)																				
**Individual HLM**										**Pooled HLM**										
**Protein**		**Mean ± SD**			**Min–Max**		***N*** **(24)**			**Protein**				**Mean ± SD**		**Min–Max**			***N*** **(2)**	
CYP1A1		ND					24			CYP1A1				ND					2	
CYP1A2		17.7 ± 14.0			2.62–59,2		24			CYP1A2				16.9 ± 2.93		14.8–18.9			2	
CYP1B1		1.03 ± 1.59			0.34–8.4		24			CYP1B1				0.80 ± 0.12		0.72–0.89			2	
CYP2A6		50.6 ± 45.1			4.69–177		24			CYP2A6				20.9 ± 4.36		17.8–24.0			2	
CYP2B6		10.9 ± 11.2			1.01–45.3		24			CYP2B6				7.99 ± 2.12		6.5–9.5			2	
CYP2C19		5.02 ± 6.68			0.02–25.0		24			CYP2C19				4.27 ± 0.46		3.9–4.6			2	
CYP2C8		46.6 ± 39.7			7.00–162		24			CYP2C8				40.6 ± 9.15		34.1–47.0			2	
CYP2C9		127 ± 53.3			50.3–236		24			CYP2C9				132 ± 28.2		112–152			2	
CYP2D6		7.90 ± 6.24			0.00–25.1		24			CYP2D6				11.0 ± 2.88		9.0–13.1			2	
CYP2E1		92.3 ± 56.9			25.3–321		24			CYP2E1				90.4 ± 18.3		77.5–103			2	
CYP2J2		1.70 ± 0.48			1.02–2,6		24			CYP2J2				1.82 ± 0.44		1.52–2.1			2	
CYP3A4		85.8 ± 74.6			6.96–246.2		24			CYP3A4				84.1 ± 16.4		72.5–95.7			2	
CYP3A5		4.00 ± 7.66			0.31–34.2		24			CYP3A5				3.77 ± 1.28		2.86–4.7			2	
CYP3A7		2.41 ± 6.45			0.00–31.4		24			CYP3A7				1.95 ± 0.46		1.63–2.3			2	
CYP4A11		5.16 ± 2.69			1.41–1.41		24			CYP4A11				4.3 ± 0.12		4.2–4.4			2	
CYP4F2		17.1 ± 7.98			3.89–36.0		24			CYP4F2				17.0 ± 3.88		14.2–19.7			2	
Total CYP450		475 ± 39.7								Total CYP450				437 ± 39.6						
CES1		231 ± 105			24.8–491		24			CES1				226 ± 12.3		217–234			2	
CES2		16.7 ± 6.18			5.37–34.6		24			CES2				16.0 ± 0.67		15.5–16.5			2	
Total CES		248 ± 152								Total CES				242 ± 148						
**(C)**																				
**Protein**	**Duodenum**			**Proximal jejunum**					**Medial jejunum**				**Distal jejunum**					**Ileum**		
	**Mean ± SD**	**Min–Max**	***N* (4)**	**Mean ± SD**		**Min–Max**		***N* (4)**	**Mean ± SD**		**Min–Max**	**n (4)**	**Mean ± SD**		**Min–Max**		***N* (4)**	**Mean ± SD**	**Min–Max**	***N* (4)**
CYP1A1	ND		4	ND				4	ND			4	ND				4	ND		4
CYP1A2	ND		4	ND				4	ND			4	ND				4	ND		4
CYP1B1	ND		4	ND				4	ND			4	ND				4	ND		4
CYP2A6	ND		4	ND				4	ND			4	ND				4	ND		4
CYP2B6	ND		4	ND				4	ND			4	ND				4	ND		4
CYP2C19	0.53 ± 0.32	0.23–0.86	4	0.58 ± 0.24		0.30–0.82		4	0.51 ± 0.14		0.36–0.63	4	0.40 ± 0.23		0.26–0.74		4			
CYP2C8	ND		4	ND				4	ND			4	ND				4	ND		4
CYP2C9	1.99 ± 1.26	0.81–3.66	4	3.06 ± 1.33		1.30–4.45		4	2.28 ± 0.95		0.92–3.14	4	2.69 ± 1.83		0.64–4.58		4			
CYP2D6	0.22 ± 0.14	0.02–0.33	4	0.19 ± 0.19		0.00–0.46		3	0.16 ± 0.12		0.01–0.31	4	0.17 ± 0.12		0.01–0.27		4			
CYP2E1	ND		4	ND				4	ND			4	ND				4	ND		4
CYP2J2	0.15 ± 0.08	0.05–0.22	4	0.15 ± 0.05		0.08–0.20		4	0.24 ± 0.09		0.14–0.32	4	0.22 ± 0.15		0.11–0.43		4			
CYP3A4	3.68 ± 2.4	0.93–6.77	4	5.55 ± 7.15		0.75–16.1		4	8.89 ± 10.7		2.85–24.9	4	4.09 ± 4.04		1.78–10.1		4			
CYP3A5	0.39 ± 0.23	0.11–0.64	4	0.29 ± 0.15		0.15–0.45		4	0.31 ± 0.23		0.14–0.31	4	0.23 ± 0.13		0.13–0.42		4			
CYP3A7	ND		4	ND				4	ND			4	ND				4	ND		4
CYP4A11	ND		4	ND				4	ND			4	ND				4	ND		4
CYP4F2	1.3 ± 1.06	0.50–2.79	4	1.9 ± 1.2		0.82–3.47		4	3.11 ± 2.61		0.27–6.0	4	2.28 ± 1.75		0.54–4.68		4			
Total CYP450	8.25 ± 1.16			11.7 ± 1.78					15.5 ± 2.69				10.07 ± 1.42					14.58 ± 2.45		
CES1	1.51 ± 0.87	0.33–2.21	4	0.60 ± 0.24		0.36–0.91		4	0.61 ± 0.21		0.43–0.82	4	0.40 ± 0.16		0.29–0.64		4			
CES2	37.73 ± 32.4	9.49–83.0	4	36.1 ± 27.6		9.18–69.8		4	34.9 ± 23.0		12.5–66.7	4	28.6 ± 18.0		5.11–44.5		4			
Total CES	39.2 ± 25.6			36.7 ± 25.1					35.5 ± 24.3				28.9 ± 19.9					21.4 ± 14.7		

**Table 3:** Spearman’s correlation analysis between CYP450 activities and CYP450 content in the following: (**A**) Individual human hepatocytes, (**B**) genotyped human liver microsomes, and (**C**) cryopreserved human intestinal mucosa.

(**A**)				
**CYP450**	**Probe**	**r**	***p***	***N***
CYP1A2	Phenacetin	0.788	<0.0001	20
CYP2A6	Coumarin	0.7985	<0.0001	20
CYP2B6	Bupropion	0.7424	0.0002	20
CYP2C8	Amodiaquine	0.7574	0.0001	20
CYP2C9	Diclofenac	0.3805	0.098	20
CYP2C19	Mephenytoin	0.7003	0.0006	20
CYP2D6	Dextromethorphan	0.8015	<0.0001	20
CYP2E1	Chlorzoxazone	0.2915	0.2124	20
CYP3A4	Midazolam	0.5353	0.015	20
CYP3A4	Testosterone	0.7684	<0.0001	20
(**B**)				
**CYP450**	**Probe**	**r**	***p***	***N***
CYP1A2	Phenacetin	0.6814	0.0046	16
CYP2A6	Coumarin	0.9471	<0.0001	16
CYP2B6	Bupropion	0.9794	<0.0001	16
CYP2C8	Amodiaquine	0.9176	<0.0001	16
CYP2C9	Diclofenac	0.65	0.0078	16
CYP2C19	Mephenytoin	0.95	<0.0001	16
CYP2D6	Dextromethorphan	0.8291	0.0002	16
CYP2E1	Chlorzoxazone	0.9441	<0.0001	16
CYP3A4	Midazolam	0.8168	0.0002	16
CYP3A4	Testosterone	0.9559	<0.0001	16
CYP4A11	Lauric Acid	0.7176	0.0024	16
(**C**)				
**CYP450/Enzyme**	**Probe**	**r**	***p***	***N***
CYP2C9	Diclofenac	−0.3071	0.265	15
CYP2C19	Mephenytoin	0.7036	0.0045	15
CYP2D6	Dextromethorphan	0.5771	0.0266	15
CYP3A4	Midazolam	0.1321	0.6389	15
CYP3A4	Testosterone	0.2321	0.4039	15
CYP2J2	Astemizole	0.1679	0.5492	15
CES2	Irinotecan	−0.0643	0.8225	15

## Data Availability

The original contributions presented in this study are included in the article/Supplementary Materials. Further inquiries can be directed to the corresponding author.

## Supplementary Materials

The following supporting information can be downloaded at: https://www.mdpi.com/article/10.3390/ph18121789/s1, Figure S1: CYP450 and CES expression in Cryopreserved human intestinal mucosa (CHIM) sections; Figure S2: Human hepatocyte (HHEP) activity and correlation with enzyme content; Figure S3: Human liver microsome (HLM) activities and correlation with enzyme content; Figure S4: Cryopreserved human intestinal mucosa (CHIM) activities and correlation with enzyme content. Table S1: CYP2C9, CYP2C19, CYP2D6, and CYP3A5 genotype for Human Liver Microsomes. Table S2: Proteospecific peptides sequences, MRM transitions, Collision Energy (CE) used for MS quantification; Table S3: Intra-day (six replicates) and inter-day (three individuals runs) precision and accuracy results for proteotypic peptides in ammonium bicarbonate; Table S4: Matrix effect results in 6 different self-prepared human intestine microsomes; Table S5: Autosampler stability results.

## Abbreviations

The following abbreviations are used in this manuscript:

A	Asian
AA	African-American
ACN	Acetonitrile
AN	Anoxia
AS	American Samoan
BSA	Bovine serum albumin
C	Caucasian
CES	Carboxyl esterase
CHIM	Cryopreserved human intestinal mucosa
CVA	Cerebrovascular accident
CYP450	Cytochrome P450
DDT	Dithiothreitol
F	Female
FA	Formic acid
GSW	Gunshot wound
H	Hispanic
H_2_O	Water
HHEP	Human hepatocytes
HLM	Human liver microsomes
HPLC/MS/MS	High-performance liquid chromatography/tandem mass spectrometry
HT	Head trauma
IAA	Iodoacetamide
LLOQ	Lower limit of quantification
M	Male
MPER	Mammalian protein extraction reagent
ND	Not detected
PBS	Phosphate-buffered saline
QC	Quality control
SD	Standard deviation
TFA	Trifluoroacetic acid
TPER	Tissue protein extraction reagent

## Appendix A. Supplementary Methods: CES1 and CES2 Quantification Method Validation

Two proteotypic peptides were selected for CES1 (ELIPEATEK and YLGGTDDTVK) and CES2 (ADHGDELPFVFR and IQELEEPEER). Stable isotope-labeled internal standards, with 13C-labeled lysine (K) or 15N-labeled arginine (R) in C-terminal, were synthesized for all peptides. The amino acid sequences of the proteotypic peptides and their internal standard are reported in Table S1. The CES1 and CES2 quantification method was validated according to the current U.S. FDA guidelines for bioanalytical methods, as no guidelines for proteomics assays have yet been developed [35]. The assay was linear over a range of 0.1 to 15 nM, and a linear regression (1/X) produced the best fit for the concentration–detector relationship. The correlation coefficients (r^2^) were greater than 0.9949, the lower limit of quantification (LLOQ) precision was better than 14.9%, and the LLOQ accuracy was between 86.9 and 115.7%. The intra-day (n = 6) and the inter-day (n = 18) precision and accuracy were assessed by replicate analyses of quality control (QC) samples at three concentrations (low, mid, and high) within an analytical run or within three independent analytical runs, respectively. For these QC samples, the precision was better than 11.6% and the accuracy was in the 91.2–103.5% range. The precision and accuracy results are shown in Supplementary Table S2. Matrix effect and autosampler stability were evaluated by back-calculating QC concentrations against the calibration curve. Matrix effect was evaluated in six lots of digested HIM fortified with mid QC, high QC or ACN:H_2_O solution (40:60, *v*/*v*) in triplicate. Autosampler stability was assessed by keeping low-, mid-, and high-QC samples in the autosampler for 98 h at 15 °C. Percent nominal was between 84.4 and 114.7% and 99.1 and 119.0% for matrix effect (Supplementary Table S3) and autosampler stability (Supplementary Table S4), respectively. For each CYP450, protein quantification was measured with two proteotypic peptides. Final CYP450 protein amount was calculated as the average of these two measured peptides. Correlation analyses between the two proteotypic peptides were assessed using six different lots of digested HIM and were evaluated using the Spearman rank test. Strong correlations were observed (r > 0.8286).
