# Supplementary material for: Protein Levels of 16 Cytochrome P450s and 2 Carboxyl Esterases Using Absolute Quantitative Proteomics: CYP2C9 and CYP3A4 Are the Most Abundant Isoforms in Human Liver and Intestine, Respectively
Source: Pharmaceuticals (Basel). 2025 Nov 25;18(12):1789. doi: 10.3390/ph18121789 (PMC12735975; doi:10.3390/ph18121789)
Supplement: Supplementary file 1 [file pharmaceuticals-18-01789-s001.zip › pharmaceuticals-3959772-supplementary.pdf]

Supplementary Table S1. CYP2C9, CYP2C19, CYP2D6 and CYP3A5 genotype for Human Liver Microsomes.

Subject	CYP2C9	CYP2C19	CY2D6	CYP3A5
HLM01	2C9*2/*3	2C19*1/*1	2D6 *1/*4	Unknown
HLM02	2C9*1/*2	2C19*1/*1	2D6 *2/*3	Unknown
HLM03	2C9*1/*3	2C19*1/*1	2D6 *1/*4	Unknown
HLM04	2C9*1/*1	2C19*1/*2	CYP2D6*1/*41	3A5*3/*3
HLM05	2C9*1/*1	2C19*1/*2	CYP2D6*4/*41	Unknown
HLM06	2C9*1/*1	2C19*1/*2	CYP2D6*2/*2	3A5*3/*3
HLM07	2C9*1/*1	2C19*2/*2	CYP2D6*4/*4	3A5*3/*3
HLM08	2C9*1/*1	2C19*1/*2	2D6*4/*6	Unknown
HLM09	2C9*1/*2	2C19*1/*2	2D6*4/*4	3A5*3/*3
HLM10	2C9*1/*1	2C19*1/*1	2D6*4/*5	3A5*3/*3
HLM11	Unknown	Unknown	Unknown	3A5*3/*3
HLM12	Unknown	Unknown	Unknown	3A5*3/*3
HLM13	Unknown	Unknown	Unknown	3A5*3/*3
HLM14	Unknown	Unknown	Unknown	3A5*3/*3
HLM15	Unknown	Unknown	Unknown	3A5*3/*3
HLM16	Unknown	Unknown	Unknown	3A5*3/*3

Supplementary Table S2. Proteospecific peptides sequences, MRM transitions, Collision Energy (CE) used for MS quantification. The internal standards are labelled with lysine or arginine terminal (K^ or R^).

Protein	Proteotypic peptide	MWs	Precursor (m/z)	z	Product (m/z)	Ion/z	CE (eV)	Product (m/z)	Ion/z	CE (eV)	Product (m/z)	Ion/z	CE (eV)
CES1	ELIPEATEK	1028.5	515.3	2+	674.5	y6/1+	14.7	243.1	b2/1+	17.6	787.4	y7/1+	16.8
	ELIPEATEK^	1036.5	519.3	2+	682.5	y6/1+	14.7	243.1	b2/1+	17.6	795.4	y7/1+	16.8
	YLGGTDDTVK	1067.5	534.8	2+	792.4	y8/1+	17.0	277.2	b2/1+	17.2			
	YLGGTDDTVK^	1075.5	538.8	2+	800.4	y8/1+	17.0	277.2	b2/1+	17.2			
CES2	ADHGDELPFVFR	1401.7	468.2	3+	333.2	y5/2+	16.9	665.3	y5/1+	13.5	738.4	b7/1+	13.9
	ADHGDELPFVFR^	1411.7	471.6	3+	338.2	y5/2+	16.9	675.3	y5/1+	13.5	738.4	b7/1+	13.9
	IQELEEPEER	1270.6	636.3	2+	530.2	y4/1+	14.5	1030.4	y8/1+	17.2	242.2	b2/1+	16.5
	IQELEEPEER^	1280.6	641.3	2+	540.2	y4/1+	14.5	1040.2	y8/1+	17.2	242.2	b2/1+	16.5

Supplementary Table S3. Intra-day (six replicates) and inter-day (three individuals runs) precision and accuracy results for proteotypic peptides in ammonium bicarbonate. Results are expressed as concentration (mean ± SD). RSD is for Relative Standard Deviation and RE for Relative Error.

Protein	Conc.	Peptide	Intra-day (n=6)			Inter-day (n=18)		
	(nM)		Mean ± SD (nM)	RSD (%)	RE (%)	Mean ± SD (nM)	RSD (%)	RE (%)
CES1	0.1	ELIPEATEK	0.092 ± 0.00	3.4	8.2	0.090 ± 0.01	8.3	10.3
	0.5		0.54 ± 0.02	4.2	-8.8	0.52 ± 0.03	5.1	-4.2
	5		5.0 ± 0.17	3.4	-1.1	5.2 ± 0.24	4.6	-3.3
	15		14.5 ± 0.28	2.0	3.5	15.1 ± 1.1	7.2	-0.8
CES1	0.1	YLGGTDDTVK	0.084 ± 0.01	10.0	15.7	0.086 ± 0.01	10.0	14.4
	0.5		0.50 ± 0.03	6,1	-0.27	0.51 ± 0.03	5.6	-1.2
	5		5.0 ± 0.20	4	-0.77	5.1 ± 0.22	4.3	-2.4
	15		15.0 ± 0.82	5,5	0.27	14.7 ± 1.1	7.6	1.8
CES2	0.1	ADHGDELPFVFR	0.099 ± 0.01	5,9	0.78	0.10 ± 0.01	12.5	-0.55
	0.5		0.52 ± 0.03	9,1	-4.8	0.51 ± 0.04	7.9	-2.3
	5		5.1 ± 0.10	1,2	-1.3	5.1 ± 0.20	4.0	-1.6
	15		15.1 ± 0.62	4,1	-0.93	14.9 ± 1.1	7.0	0.54
CES2	0.1	IQELEEPEER	0.098 ± 0.01	8.6	1.6	0.11 ± 0.03	14,9	-13.1
	0.5		0.53 ± 0.02	3.9	-5.4	0.54 ± 0.06	11,6	-8.7
	5		5,0 ± 0.27	5.4	0.8	5.1 ± 0.30	5.9	-1.5
	15		14,9 ± 0.49	3.3	0.55	14.9 ± 1.2	8,1	0.73

Supplementary Table S4. Matrix effect results in 6 different self-prepared human intestine microsomes. Results are expressed as percent nominal ± SD.

Protein	Peptide	Concentration	%Nominal (mean ± SD, n=3)					
		(nM)	Matrice 1	Matrice 2	Matrice 3	Matrice 4	Matrice 5	Matrice 6
CES1	ELIPEATEK	5	104.8 ± 0.6	108.3 ± 5.5	97.7 ± 1.1	98.5 ± 2.3	114.4 ± 0.2	105.2 ± 8.8
		15	110.7 ± 4.8	114.7 ± 7.6	104.9 ± 0.2	108.9 ± 7.6	100.3 ± 1.9	103.4 ± 6.0
	YLGGTDDTVK	5	89.1 ± 3.8	95.0 ± 3.9	89.3 ± 13.6	90.9 ± 4.5	110.6 ± 4.6	94.8 ± 0.5
		15	98.6 ± 3.9	97.2 ± 2.0	89.4 ± 8.2	95.4 ± 0.6	87.4 ± 7.1	91.9 ± 0.8
CES2	ADHGDELPFVFR	5	90.3 ± 10.1	96.3 ± 12.3	97.5 ± 17.7	93.7 ± 1.3	109.8 ± 20.2	97.9 ± 12.7
		15	108.6 ± 15.6	104.4 ± 4.7	105.1 ± 5.2	105.4 ± 0.7	84.4 ± 6.6	96.4 ± 4.0
	IQELEEPEER	5	88.6 ± 10.0	97.4 ± 7.6	88.9 ± 14.9	111.8 ± 20.8	98.1 ± 8.3	103.8 ± 0.3
		15	107.2 ± 9.3	111.9 ± 2.7	89.5 ± 12.5	111.7 ± 14.1	89.0 ± 0.9	99.5 ± 5.1

Supplemental Table S5. Autosampler stability results. Results are expression as percent nominal ± SD.

Protein	Peptide	Conc.	Autosampler (98h, 15˚C), n=6	Peptide	Conc.	Autosampler (98h, 15˚C), n=6
		(nM)	%Nominal (mean ± SD)		(nM)	%Nominal (mean ± SD)
CES1	ELIPEATEK	0.5	106,3 ± 6,2	YLGGTDDTVK	0.5	108,6 ± 5,6
		5	101,2 ± 1,7		5	102,2 ± 1,5
		15	104,3± 1,4		15	101,7 ± 4,2
CES2	ADHGDELPFVFR	0.5	99,1 ± 4,1	IQELEEPEER	0.5	119,0 ± 3,6
		5	100,8 ± 0,04		5	107,3 ± 3,7
		15	106,4 ± 2,0		15	106,4 ± 3,6

Supplementary Figure S1

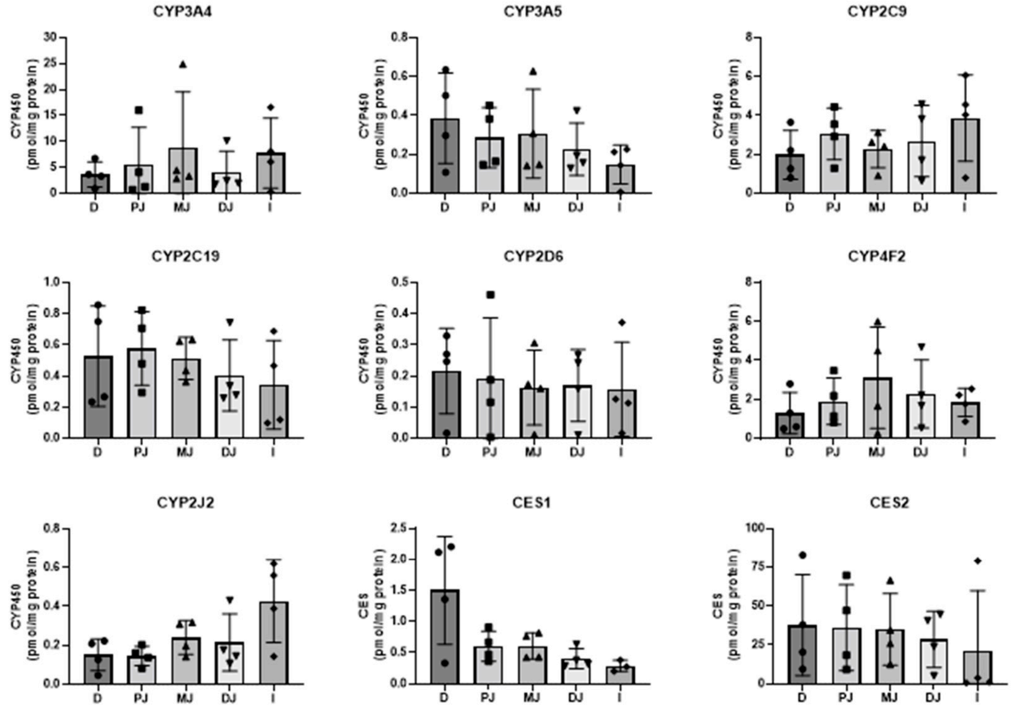

Supplemental Figure S1: CYP450 and CES expression in Cryopreserved human intestinal mucosa (CHIM) sections. These data are the same from Figure 2 but re-graphed with each chart representing a single CYP450 or CES enzyme. Duodenum (D), proximal jejunum (PJ), medial jejunum (MJ), distal jejunum (DJ) and ileum (I). Data are displayed as mean ± standard deviation.

Figure S2

A

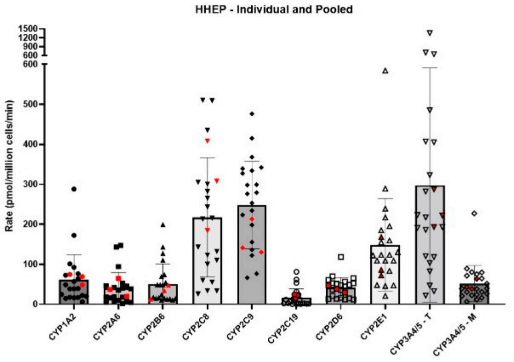

Mean ± SD	Individual HHEP (n = 20)	Pooled HHEP (n = 3)
CYP1A2	61.2 ± 66.5	63.9 ± 13.8
CYP2A6	39.5 ± 42.5	40.2 ± 22.4
CYP2B6	53.5 ± 53.2	31.4 ± 17.4
CYP2C8	204.6 ± 152.0	300.0 ± 112.2
CYP2C9	260.8 ± 110.4	161.3 ± 45.1
CYP2C19	15.8 ± 23.3	20.6 ± 5.3
CYP2D6	42.0 ±26.6	38.1 ± 9.0
CYP2E1	154.6 ± 122.2	108.6 ± 51.9
CYP3A4/5	306.9 ± 313.9 (Testosterone) 52.2 ± 48.3 (Midazolam)	234.0 ± 48.1 (Testosterone) 48.2 ± 13.4 (Midazolam)

B

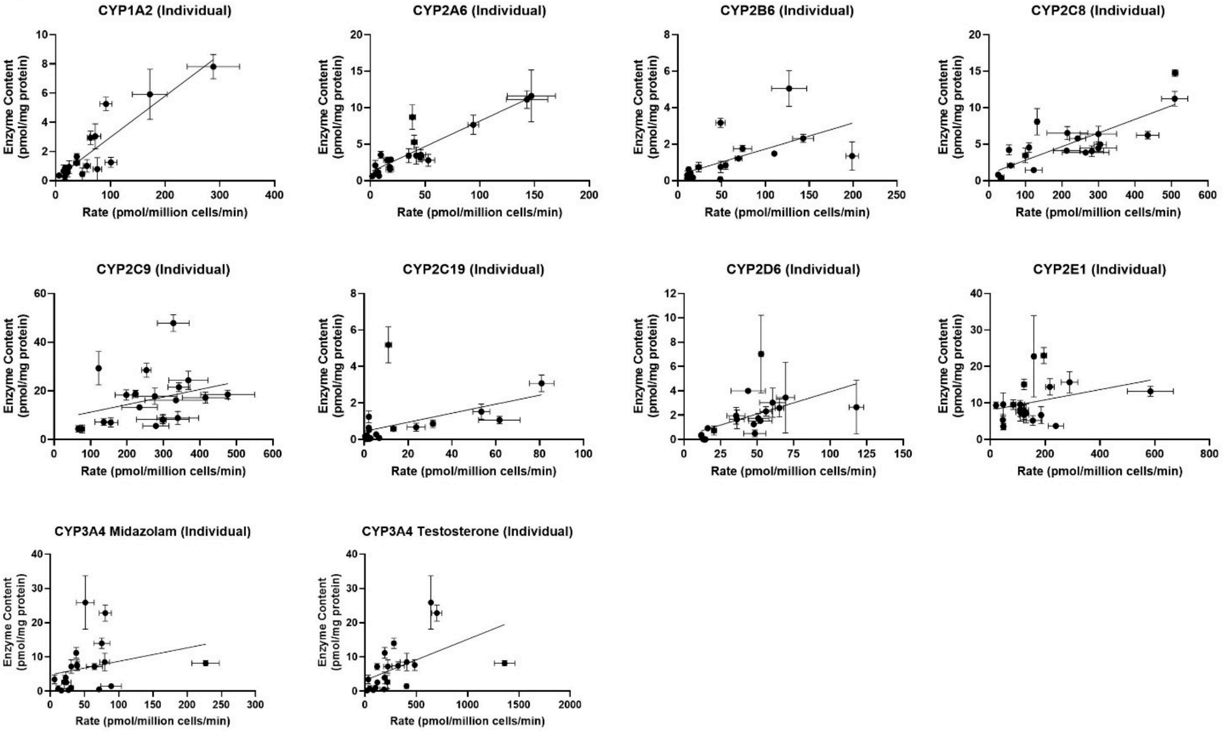

Figure S2: Human hepatocyte (HHEP) activities and correlation with enzyme content. (A) Left – HHEP enzyme activity data are displayed. The black dots represent the individual HHEP samples, and the red dots represent the pooled HHEP samples. CYP3A4/5 – T (Testosterone), CYP3A4/5 – M (Midazolam). Right – the table displays the mean ± standard deviation activity values for each CYP450 from the individual or pooled HHEP samples separately. (B) The individual enzyme content from Table 2A and the individual enzyme activity from (A) above are displayed on a scatter plot. Data are displayed as mean ± standard deviation. A simple linear regression (B) was performed for statistical analysis.

Figure S3

A

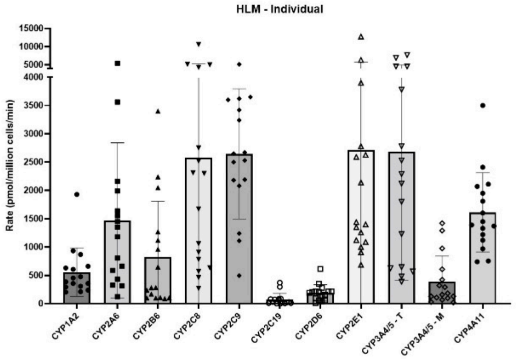

Mean ± SD	Individual HLM (n = 16)
CYP1A2	557.2 ± 427.7
CYP2A6	1470.7 ± 1368.7
CYP2B6	926.5 ± 1009.6
CYP2C8	2580.1 ± 2668.0
CYP2C9	2644.7 ± 1148.4
CYP2C19	230.3 ± 183.3
CYP2D6	253.3 ± 131.9
CYP2E1	2715.3 ± 3049.9
CYP3A4/5	2684.6 ± 2274.8 (Testosterone)<br>503.1 ± 470.8 (Midazolam)
CYP4A11	1612.1 ± 699.1

B

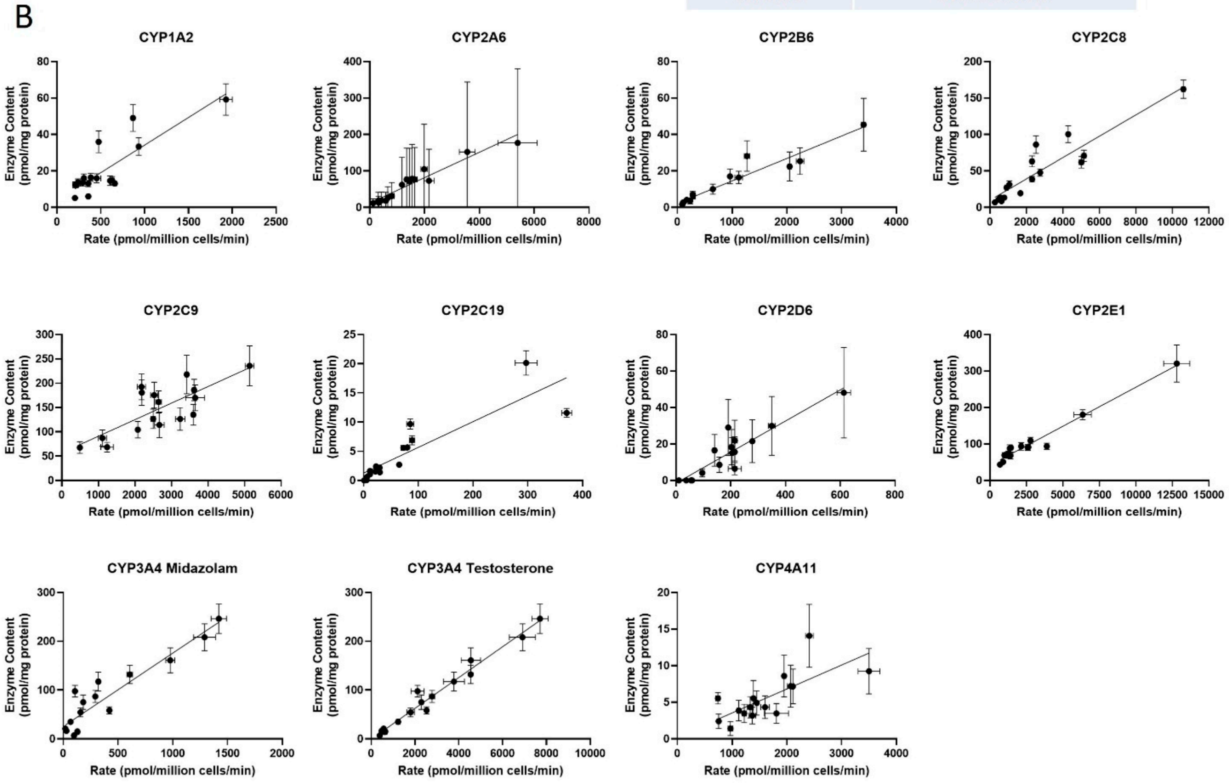

Figure S3: Human liver microsome (HLM) activities and correlation with enzyme content. (A) Left – HLM individual enzyme activity data are displayed. CYP3A4/5 – T (Testosterone), CYP3A4/5 – M (Midazolam). Right – the table displays the mean ± standard deviation activity values for each CYP450 from the individual HLM samples. (B) The individual enzyme content from Table 2A and the individual enzyme activity from (A) above are displayed on a scatter plot. Data are displayed as mean ± standard deviation. A simple linear regression (B) was performed for statistical analysis.

Figure S4

A

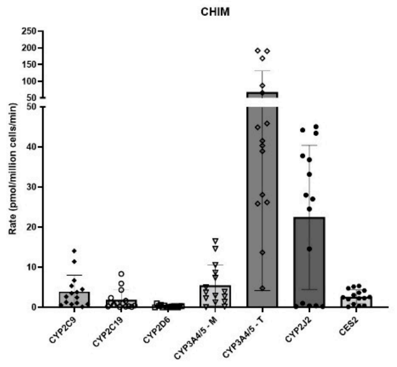

Mean ± SD	Individual CHIM (n = 15)
CYP2C9	3.9 ± 4.1
CYP2C19	1.9 ± 2.5
CYP2D6	0.2 ± 0.3
CYP3A4/5	5.4 ± 5.1(Midazolam)<br>67.7 ± 63.5 (Testosterone)
CYP2J2	22.5 ± 18.0
CES2	2.6 ± 1.7

B

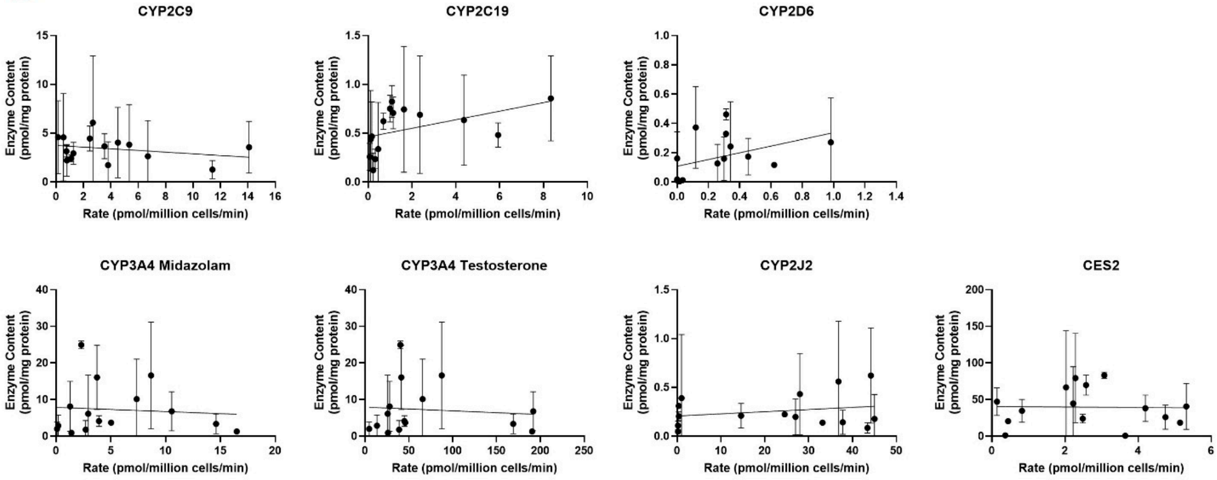

Figure S4: Cryopreserved human intestinal mucosa (CHIM) activities and correlation with enzyme content. (A) Left – CHIM individual enzyme activity data are displayed. Three donors were utilized with 5 samples from each representing one intestinal section - duodenum, proximal jejunum, medial jejunum, distal jejunum and ileum (n = 3 patients x 5 samples per patient = 15 total). CYP3A4/5 – M (Midazolam), CYP3A4/5 – T (Testosterone). Right – the table displays the mean ± standard deviation activity values for each CYP450 from the individual CHIM samples. (B) The individual enzyme content from Table 2C and the individual enzyme activity from (A) above are displayed on a scatter plot. Data are displayed as mean ± standard deviation. A simple linear regression (B) was performed for statistical analysis.
